# Correction: Yamamoto et al. Intestinal Morphology and Glucose Transporter Gene Expression under a Chronic Intake of High Sucrose. *Nutrients* 2024, *16*, 196

**DOI:** 10.3390/nu17172789

**Published:** 2025-08-28

**Authors:** Kana Yamamoto, Norio Harada, Takuma Yasuda, Tomonobu Hatoko, Naoki Wada, Xuejing Lu, Yohei Seno, Takashi Kurihara, Shunsuke Yamane, Nobuya Inagaki

**Affiliations:** 1 Department of Diabetes, Endocrinology and Nutrition, Graduate School of Medicine, Kyoto University, Kyoto 606-8507, Japan; 2 P.I.I.F. Tazuke-Kofukai Medical Research Institute, Kitano Hospital, Osaka 530-8480, Japan

## Author Name Change

In the original publication [1], the spelling of one of the co-authors’ names, “Youhei Seno” was wrong. It should be spelled as “Yohei Seno”, so that he can secure the academic credit he deserves.

## Text Correction

There was an error in the original publication, in Materials and Methods, Section 2.1, paragraph 2. While the concentration of “miglitol” was listed as “0.008%”, the actual concentration is “0.08%”.

## Error in Figure

There were errors in the plots in Figure 5A iAUC-glucose and Supplementary Figure S4A AUC-glucose. The positions of the plots were incorrect due to an unintentional error during the editing process. The corrected Figure 5A iAUC-glucose and Supplementary Figure S4A AUC-glucose appear below.

Figure 5A iAUC-glucose:


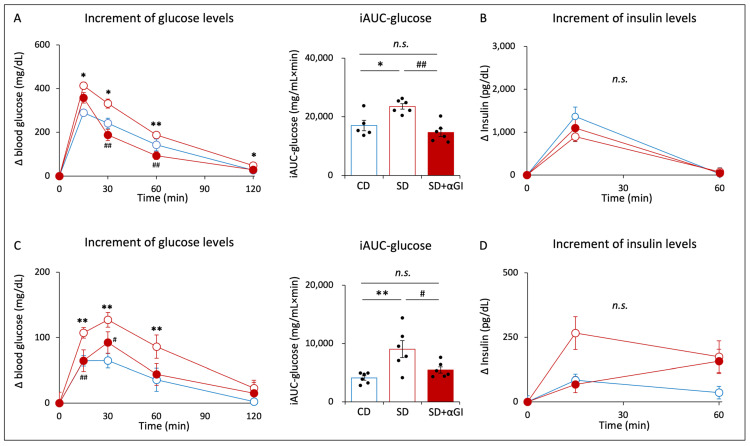


Supplementary Figure S4A AUC-glucose:


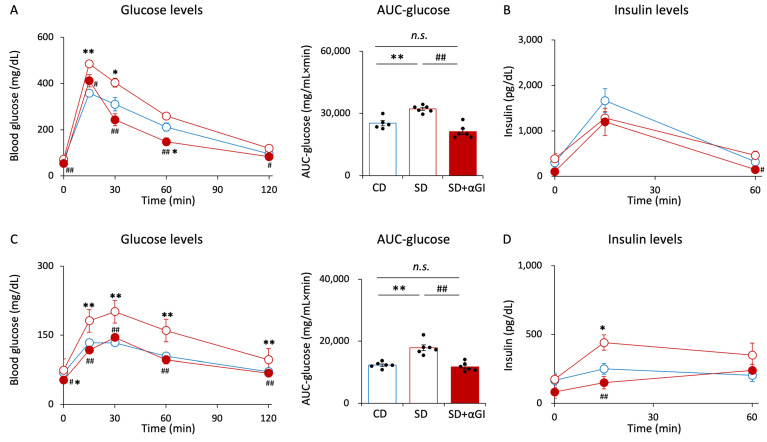



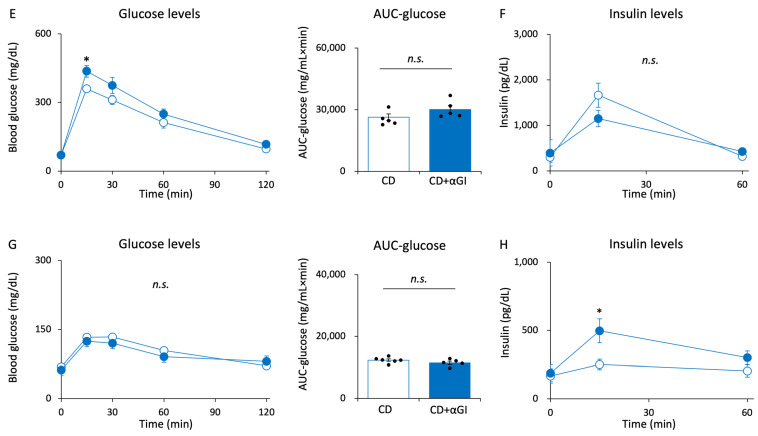


In addition, the authors state that the scientific conclusions are unaffected. These corrections were approved by the Academic Editor. The original publication has also been updated.
