# Supplementary material for: Intestinal Morphology and Glucose Transporter Gene Expression under a Chronic Intake of High Sucrose
Source: Nutrients. 2024 Jan 7;16(2):196. doi: 10.3390/nu16020196 (PMC10820040; doi:10.3390/nu16020196)
Supplement: Supplementary file 1 [file nutrients-16-00196-s001.zip › nutrients-2797429-supplementary.pdf]

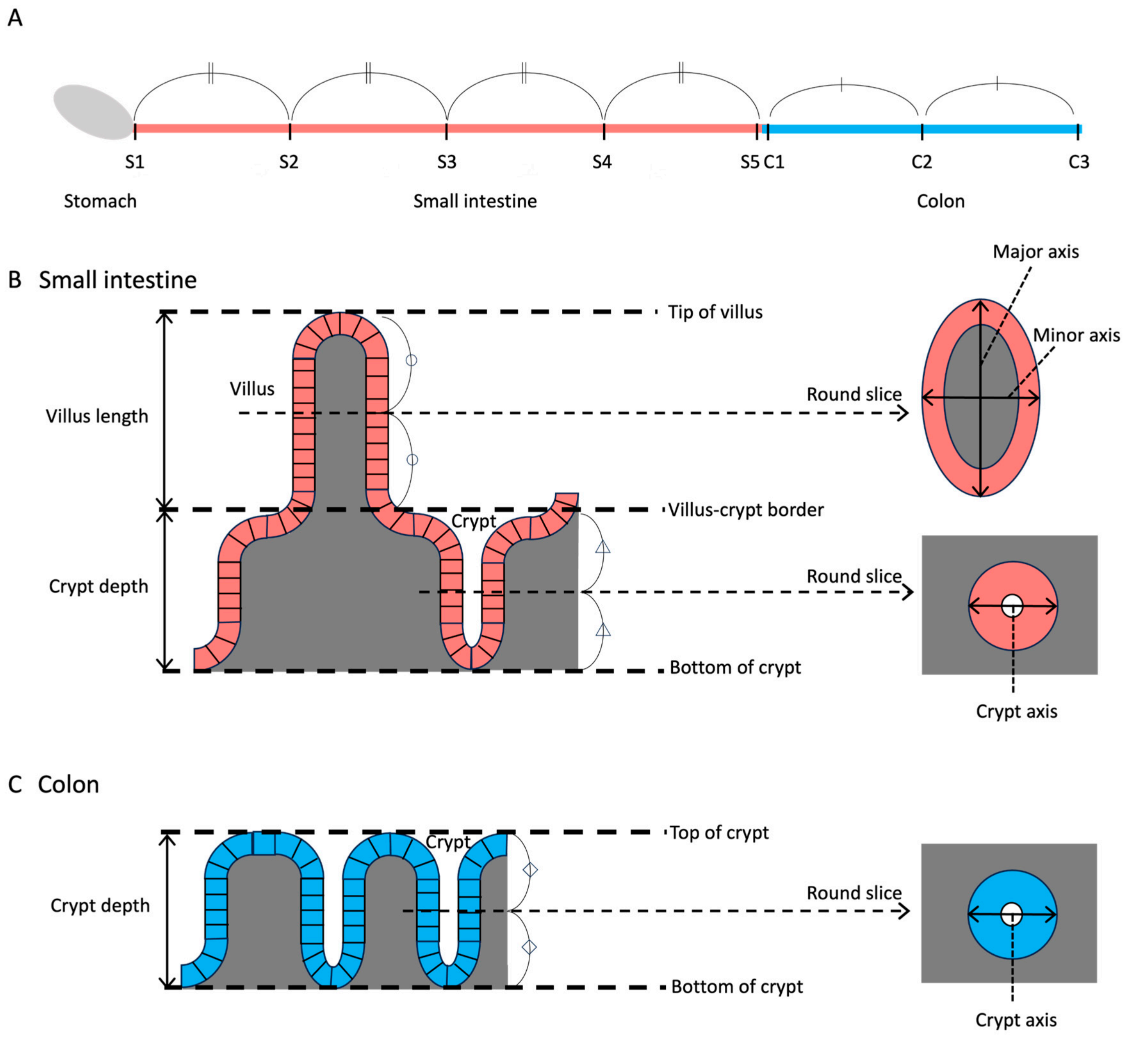

Supplementary Figure S1: Intestinal samples and measuring method.

(A) Five sections of small intestine (S1 to S5) and three sections of colon (C1 to C3) were analyzed for intestinal morphology and mRNA expression of small intestine. (B) Measurement of villus length, major axis, minor axis, crypt depth and crypt axis in small intestine. (C) Measurement of crypt depth and crypt axis in colon.

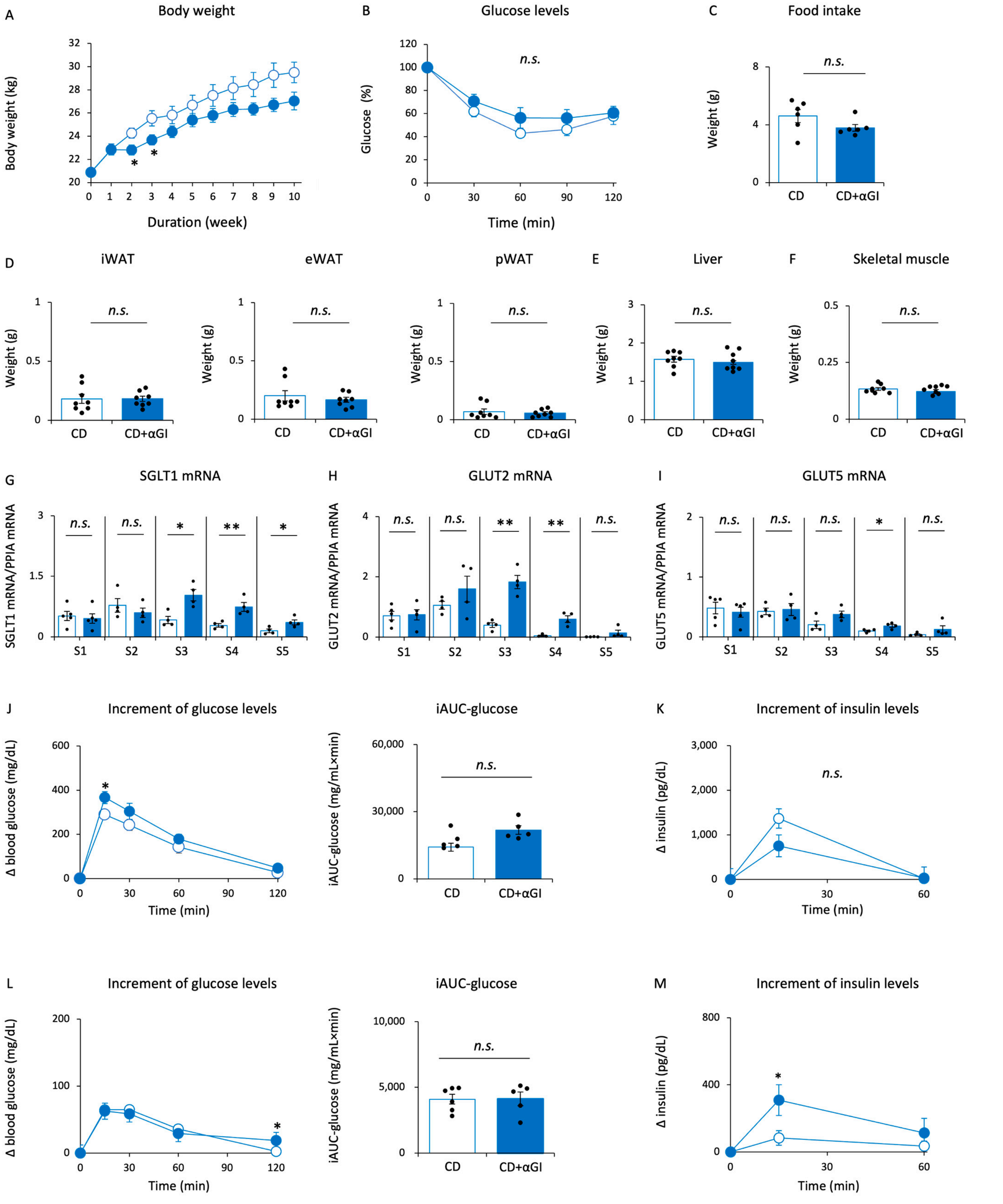

Supplementary Figure S2: Phenotype, the mRNA expression of transporters, tolerance tests of CD mice and CD+αGI mice.

Comparison of CD mice and CD+αGI mice. CD mice are represented by white circles with blue frames and blue boxes. CD+αGI mice are represented by blue circles and blue bars. (A) Body weight (n = 6). (B) Insulin tolerance test (n = 6). (C) Food intake for 24 hours (n = 6). Weight of (D) inguinal white adipose tissue (iWAT), epididymal WAT (eWAT), and perirenal WAT (pWAT), (E) liver, and (F) skeletal muscle (n = 8).

mRNA expression levels of (G) SGLT1, (H) GLUT2, and (I) GLUT5 mRNA in small intestine (n = 4-5). $^{*}P < 0.05$, $^{**}P < 0.01$. Oral glucose tolerance test (OGTT) and oral fructose tolerance test (OFTT) of CD mice and CD+αGI mice. CD mice are represented by white circles with blue frames and blue boxes. CD+αGI mice are represented by blue circles and blue bars. iAUC indicates incremental area under the curve. (J) The increment of blood glucose levels and (K) the increment of insulin levels during OGTT (n = 5). (L) The increment of blood glucose levels and (M) the increment of insulin levels during OFTT (n = 5-6). $^{*}P < 0.05$, $^{**}P < 0.01$ vs. CD. *n.s.*: not significant.

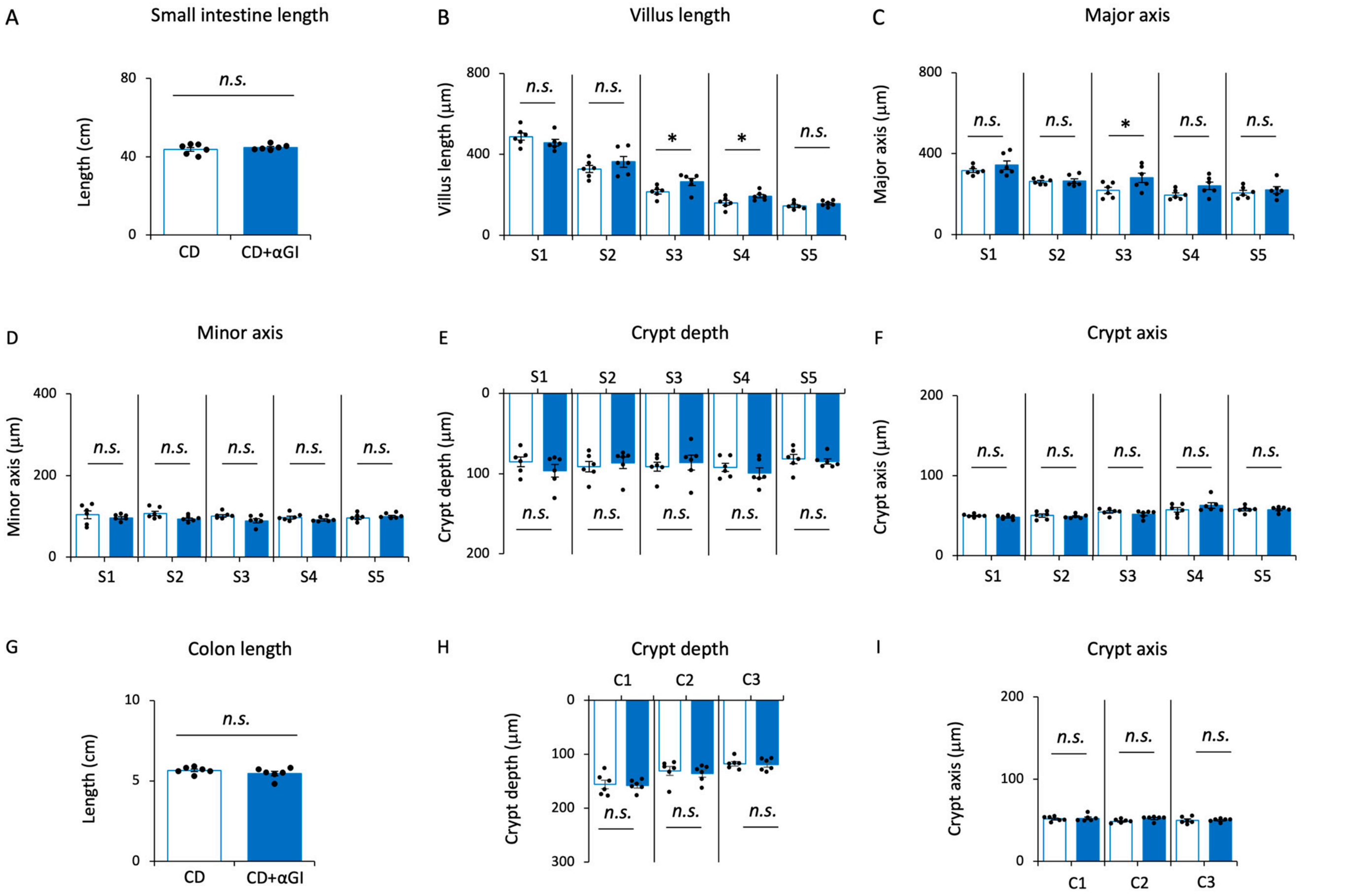

Supplementary Figure S3: Morphology of the intestine of CD mice and CD+αGI mice.

Length of (A) small intestine (n = 6). (B) Villus length, width of (C) villus major axis, (D) villus minor axis, (E) crypt depth, and (F) crypt axis of small intestine. Length of (G) colon (n = 6). (H) Crypt depth and (I) crypt axis of colon (n = 6). $^{*}P < 0.05$, $^{**}P < 0.01$. *n.s.*: not significant.

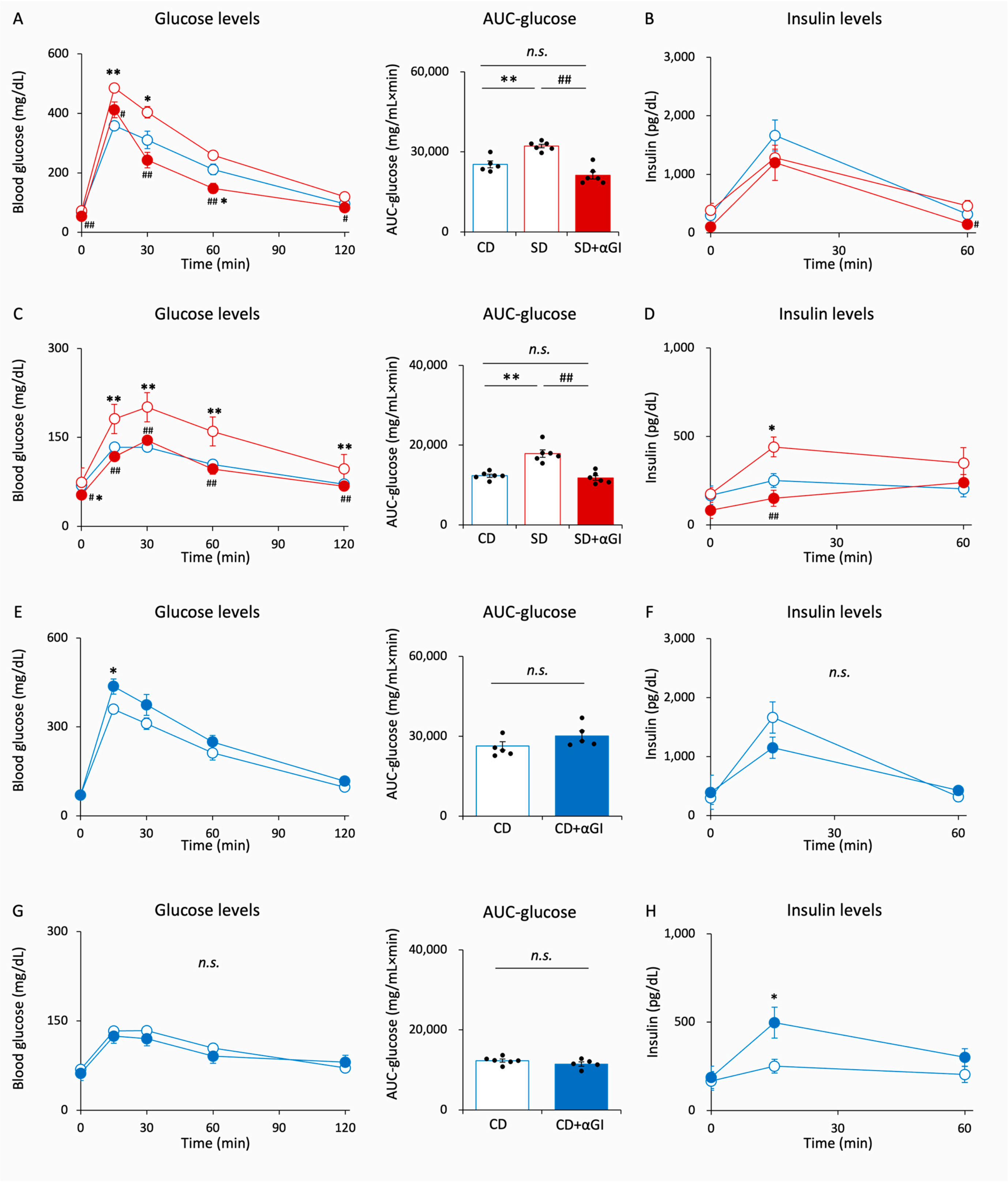

Supplementary Figure S4: Uncorrected data of glucose and fructose tolerance tests.

The absolute values of blood glucose and insulin levels during oral glucose tolerance tests (OGTTs) and oral fructose tolerance tests (OFTTs). (A-D) Comparison of CD mice, SD mice, and SD+αGI mice. CD mice are represented by white circles with blue frames and blue boxes. SD mice are represented by white circles with red frames and red boxes.

SD+αGI mice are represented by red circles and red bars. AUC indicates area under the curve. (A) Blood glucose levels and (B) insulin levels during OGTT (n = 5-6). (C) Blood glucose levels and (D) insulin levels during OFTT (n = 6). (E-H) Comparison of CD mice and CD+αGI mice. CD mice are represented by white circles with blue frames and blue boxes. CD+αGI mice are represented by blue circles and blue bars. AUC indicates area under the curve. (E) Blood glucose levels and (F) insulin levels during OGTT (n = 5). (G) Blood glucose levels and (H) insulin levels during OFTT (n = 5-6). *P < 0.05, **P < 0.01 vs. CD. $^{\#}P$ < 0.05, $^{\#\#}P$ < 0.01 vs. SD. *n.s.*: not significant.

	Control diet (CD)	High-sucrose diet (SD)
Energy	3.43kcal/g	3.76kcal/g
Percentage of total energy (%)		
Fat	12.8	12.8
Protein	29.2	13.6
Carbohydrates	58.0	73.6
Combination ratio of raw materials (%)		
CLEA Rodent diet CE-2	100	51.0
Sucrose	-	37.3
Cornstarch	-	6.4
Soy oil	-	2.9
Crystalline cellulose	-	2.4

Full ingredients list of CE-2

Fat	germ oil, soy oil
Protein	soybean meal, white fishmeal, yeast
Carbohydrates	wheat flour, corn, milo
Fibers	wheat bran, defatted rice bran, alfalfa meal
Vitamins	Vit. A, D3, E, B1, B2, B6, B12, C, niacin, pantothenic acid, biotin, folic acid, choline chloride, inositol
Minerals	calcium carbonate, salt, iron sulfate, manganese sulfate, cobalt sulfate, calcium iodate

Supplementary Table S1: Composition of experimental diets.

Gene name	Primer sequence (5’-3’)	
SGLT1	Forward	GTGCTGGGCTGGATATTTGT
	Reverse	AGGCCCAAGGCTAGATTGAT
GLUT2	Forward	AATGGTCGCCTCATTCTTTG
	Reverse	ATCAAGAGGGCTCCAGTCAA
GLUT5	Forward	TCATCTCTGTGTGGAAGTTG
	Reverse	AGATCTGATCGGCGTAGTAG
PPIA	Forward	GAGCTGTTTGCAGACAAAGTTC
	Reverse	CCCTGGCACATGAATCCTGG

Supplementary Table S2: Primer sequence list.
